# “A ghost doesn’t need insulin,” Cotard’s delusion leading to diabetic ketoacidosis and a body-mass index of 15: a case presentation

**DOI:** 10.1186/s12888-023-05039-6

**Published:** 2023-07-31

**Authors:** Christopher Robertson, Thomas Dunn

**Affiliations:** 1 Stella Psychiatry, Boston, USA; 2 Behavioral Health - Adolescent Outpatient, Denver Health, 723 Delaware St., Pavilion M, Denver, CO 80204 USA

**Keywords:** Cotard’s delusion, DKA, Case report, Anorexia nervosa, Diabetes Mellitus Type 1

## Abstract

**Background:**

Cotard’s Syndrome (CS) is a rare clinical entity where patients can report nihilistic, delusional beliefs that they are already dead. Curiously, while weight loss, dehydration, and metabolic derangements have been described as discussed above, a review of the literature revealed neither a single case of a severely underweight patient nor a serious metabolic complication such as Diabetic Ketoacidosis. Further, a search on PubMed revealed no articles discussing the co-occurrence of Cotard’s Delusion and eating disorders or comorbid metabolic illnesses such as diabetes mellitus. In order to better examine the association between Cotard’s Delusion and comorbid eating disorders and metabolic illness, we will present and discuss a case where Cotard’s delusion led to a severe metabolic outcome of DKA and a BMI of 15.

**Case presentation:**

Mr. B is a 19 year old transgender man admitted to the hospital due to diabetic ketoacidosis secondary to Type 1 Diabetes Mellitus. Mr. B had a history of Obsessive–Compulsive Disorder, Major Depressive Disorder, and Post-Traumatic Stress Disorder. The primary pediatric team discovered that Mr. B had not been using his insulin appropriately and was severely underweight, and they believed this could be due to his underlying mental illness. The psychiatric consultation/liaison service found that Mr. B was suffering from Cotard’s delusion leading him to be noncompliant with his insulin due to a belief that he was already dead. Cotard’s delusion had in this case led to a severe metabolic outcome of DKA and a BMI of 15.

**Conclusions:**

This case provides clinical insight into the interactions of eating disorders and Cotard’s delusion as well as the potential medical complications when Cotard’s delusion is co-morbid with medical conditions such as Diabetes Mellitus. We recommend that clinicians routinely screen patients for Cotard’s delusion and assess whether the presence of which could exacerbate any underlying medical illness. This includes clinicians taking special care in assessing patient’s caloric and fluid intake as well as their adherence to medications both psychiatric and medical. Further research could be conducted to explore the potential overlap of Cotard’s delusion and eating disorder phenomenology.

## Background

Cotard’s Syndrome (CS) is a rare clinical entity first described in detail in 1880 by the Parisian neurologist and psychiatrist Jules Cotard. By describing the case of a 43-year-old woman, he first characterized the syndrome as a collection of negation or nihilistic delusions, immortality delusions, and hypochondriac delusions [1, 2]. As such, patients can report delusional beliefs that they are already dead, that their organs are rotting from the inside, that nothing can harm them further as they are already dead, or that they are already suffering from an incurable disease or gross malformity [1, 2]. While he initially described the syndrome as a form of anxious melancholia [3], Cotard’s Syndrome is now more commonly conceptualized as a syndrome secondary to many other conditions both psychiatric (psychosis, mood disorders) and neurological (brain lesions, dementia) [4, 5]. Indeed, CS is not classified as an isolated disorder in the 5th edition of Diagnostic and Statistic Manual of Mental Disorders (DSM-5) neither in the International Classification of Diseases (ICD-10) [6, 7].

As Cotard’s Syndrome is a rare presentation, the literature is primarily dominated by case reports and few reviews. Along with the nihilistic and immortality delusions comes the frequent refusal of these patients to eat, drink and even accept medications described within these case reports [8–16]. These have led to metabolic complications such as hyponatremia, hypokalemia, and mild blood dyscrasias [8–11]. Curiously, while weight loss has been described, a review of the literature did not reveal a single case of a severely underweight patient nor a serious metabolic complication such as DKA. Further, a search on PubMed revealed no articles discussing the cooccurrence of Cotard’s Delusion and eating disorders or comorbid metabolic illnesses such as diabetes mellitus.

In order to better examine the association between Cotard’s Delusion and comorbid eating disorders and metabolic illness, we will present and discuss a case where Cotard’s delusion led to a severe metabolic outcome of DKA and a BMI of 15.

## Case report

Mr. B is a 19 year old transgender man admitted to the hospital due to diabetic ketoacidosis secondary to Type 1 Diabetes Mellitus. Mr. B had a history of Obsessive–Compulsive Disorder, Major Depressive Disorder, recurrent, severe without psychotic features, and Post-Traumatic Stress Disorder. The primary pediatric team discovered that Mr. B had not been using his insulin appropriately and was severely underweight, and they believed this could be due to his underlying mental illness. The psychiatry consult/liaison team was thus asked to evaluate Mr. B to address these concerns.

On interview, Mr. B reported that he had OCD and this drove a severe aversion to numbers. He had already asked his pediatric team not to tell him any numbers relevant to his care such as his blood glucose level. He also reported an aversion to needles due to his OCD. Mr. B went on to describe issues relating to his body image. Mr. B identified as a man saying that he idealizes a masculine body type. He stated his endocrinologist had already stopped his testosterone because his diabetes had not been well controlled. Mr. B reported restricting his intake leading to significant weight loss, stating that he liked the way his body looked after the weight loss and he enjoyed seeing the bones under his skin. He noted a sense of guilt associated with eating too much, though denied vomiting or other purging behaviors.

Most notably, Mr. B went on to describe his perception that he is in fact already dead consistent with Cotard’s Delusion. Mr. B reported that at the age of 16 he tried killing himself, and he believed that he had in fact succeeded and that he was a ghost. He reported that due to his belief that he was dead it didn’t feel real to him that he was a person who needed to eat, take medicine, or care for himself. Mr. B specifically described his belief that going several days without eating would not harm him as he was already deceased. He endorsed visual hallucinations of seeing skeletons and ghosts, and auditory hallucinations of overhearing conversations with dead people that occasionally seemed to address him directly. He reported delusions of reference regarding the TV and food labels, and delusions of thought broadcasting.

Throughout this interview and on subsequent interviews, Mr. B was calm and cooperative with a linear thought process, normal speech patterns, and a child-like affect. He appeared grossly underweight with a BMI of 15.46. He denied suicidal ideation though reported self-harm behaviors in his early teens consisting of cutting and burning himself and an intentional overdose of lamotrigine resulting in a psychiatric admission 3 years prior. Mr. B had a tumultuous childhood as one of many siblings in a non-intact family with a father who was frequently hospitalized psychiatrically for an unknown reason. Mr. B began to cry as he described ongoing sexual abuse by his mother and her boyfriend.

Mr. B had been being seen by a psychiatrist in the community who had diagnosed Mr. B with schizophrenia. He had been receiving treatment with aripiprazole with great effect as prior to antipsychotic initiation Mr. B was reportedly much more disorganized and delusional than he presented during his hospitalization. Ultimately, Mr. B agreed to voluntary psychiatric admission following stabilization of his DKA. The decision was made to admit Mr. B to the child and adolescent unit due to his low BMI and significant child-like presentation. He had a short stay on the child and adolescent unit and was discharged after coordination with his outpatient psychiatrist.

## Discussion

While a review of the literature suggests that an unwillingness to eat or drink or accept medications is a common presentation of Cotard’s delusion and has led to acute metabolic derangements such as hypokalemia and hyponatremia, no case reports were found suggesting 1. DKA in the setting of poorly controlled Type 1 Diabetes Mellitus and 2. Low BMI of 15.46 with body image issues. Although the paucity of cases described in the literature suggest that this presentation is extraordinarily rare, the potential lethality of this case is concerning. Indeed, Cotard’s delusion in the setting of comorbid metabolic disease of any sort should be treated with significant care as the restriction of food, fluids and medication in these tenuous, diabetic individuals could rapidly lead to serious and even fatal complications. Additionally, patients suffering with Cotard’s Syndrome chronically such as the case with Mr. B with ongoing restrictive behaviors could lead to severely underweight patients and subsequent complications.

We recommend that clinicians routinely screen patients for Cotard’s delusion and assess whether the presence of which could be exacerbating any underlying medical illness. Specifically, patients presenting with metabolic derangements or conditions secondary to insufficient caloric intake or treatment non-adherence should be screened for Cotard’s delusion as an unlikely but severe contributing factor. This includes clinicians taking special care in assessing patient’s caloric and fluid intake as well as their adherence to medications. Further research could be conducted to explore the potential overlap of Cotard’s delusion and eating disorder phenomenology.

## Data Availability

Not applicable.

## Abbreviations

CS: Cotard’s Delusion
DKA: Diabetic Ketoacidosis
BMI: Body-mass index
